# Combination of Pinocembrin and Epidermal Growth Factor Enhances the Proliferation and Survival of Human Keratinocytes

**DOI:** 10.3390/ijms241512450

**Published:** 2023-08-05

**Authors:** Jirapak Ruttanapattanakul, Nitwara Wikan, Saranyapin Potikanond, Wutigri Nimlamool

**Affiliations:** 1 Department of Pharmacology, Faculty of Medicine, Chiang Mai University, Chiang Mai 50200, Thailand; jirapak.ken@gmail.com (J.R.); nitwara.wik@cmu.ac.th (N.W.); saranyapin.p@cmu.ac.th (S.P.); 2 Graduate School, Chiang Mai University, Chiang Mai 50200, Thailand

**Keywords:** epidermal growth factor, pinocembrin, regenerative medicine, combination therapy, ERK1/2, Akt

## Abstract

Re-epithelialization is delayed in aged skin due to a slow rate of keratinocyte proliferation, and this may cause complications. Thus, there has been development of new therapies that increase treatment efficacy for skin wounds. Epidermal growth factor (EGF) has been clinically used, but this agent is expensive, and its activity is less stable. Therefore, a stable compound possessing EGF-like properties may be an effective therapy, especially when combined with EGF. The current study discovered that pinocembrin (PC) effectively synergized with EGF in increasing keratinocyte viability. The combination of PC and EGF significantly enhanced the proliferation and wound closure rate of the keratinocyte monolayer through activating the phosphorylation of ERK and Akt. Although these effects of PC were like those of EGF, we clearly proved that PC did not transactivate EGFR. Recent data from a previous study revealed that PC activates G-protein-coupled receptor 120 which further activates ERK1/2 and Akt phosphorylation. Therefore, this clearly indicates that PC possesses a unique property to stimulate the growth and survival of keratinocytes through activating a different receptor, which subsequently conveys the signal to cross-talk with the effector kinases downstream of the EGFR, suggesting that PC is a potential compound to be combined with EGF.

## 1. Introduction

Healing of skin wounds involves a series of complex processes that require many essential factors including different types of cells, growth factors, and cytokines [1,2,3]. Complete skin wound healing needs the process of re-epithelialization, in which the surrounding keratinocytes are activated to proliferate and migrate to cover the wound and maintain skin homeostasis [4]. Specifically, keratinocytes adjacent to the edge of the wound are stimulated by growth factors including transforming growth factor-beta (TGF-β), platelet-derived growth factor (PDGF), and epidermal growth factor (EGF) to divide and move along the wound bed [2,3]. However, due to aging, the proliferation rate of keratinocytes is decreased, causing a significant reduction in the regeneration capacity and suppleness of the skin [5]. Moreover, the major consequences of delayed or unsuccessful re-epithelialization are chronic wounds and persistent infections, which are considered an important health problem [6,7,8]. For this reason, many attempts have been made to promote keratinocyte proliferation and migration to increase skin regeneration capacity [9,10]. 

Numerous studies have tried to investigate the role of epidermal growth factor (EGF) in treating acute or chronic wounds. The activation of the EGF receptor (EGFR) upon EGF binding conveys the intracellular signal to activate its downstream effector kinases, including the mitogen-activated protein kinase (MAPK)/extracellular signal-regulated kinase (ERK) and phosphoinositide 3-kinase (PI3K)/Akt [11,12,13]. In turn, the activation of ERK and Akt subsequently regulates the growth, proliferation, and survival of keratinocytes [14,15,16]. Because of these pivotal roles of EGF, exogenous bioidentical human recombinant epidermal growth factor (rhEGF) has been clinically used to treat various wound types [17]. Although EGF has been proved to be effective for promoting wound healing, there are many reasons that reduce its efficacy in the actual clinical setting. For example, the wound exudate makes it difficult to apply topical EGF ointment, and EGF is a peptide that can be degraded by biofilm proteases [18]. In addition, since EGF is a peptide where the stability of the growth factor can be altered by environmental factors, it is challenging to conserve and control the intact physiological activity of EGF [19]. Furthermore, the molecular size of EGF is too large to allow it to pass the skin layer [20]. Importantly, the cost of producing synthesized EGF is high; thus, pure human EGF is expensive, making it less accessible to many patients [19,21]. Based on these limitations of EGF, there have been attempts to identify and characterize many naturally derived compounds that can transactivate EGFR but possess better cellular stability than that of EGF [22,23].

Many plants in the Zingiberaceae family have been reported to exhibit a variety of pharmacological activities (related to the regulation of the cell growth, survival, transformation, and inflammatory signaling) [24,25,26,27,28,29,30], including wound-healing properties [31,32,33]. Pinocembrin (PC) is a flavonoid (with different pharmacological activities) prevalent in many plants including *Boesenbergia rotunda* and in natural products such as Calabrian honeys [34,35,36,37]. In particular, we previously reported that PC stimulated human keratinocyte growth and survival through activating ERK1/2 and Akt [38]. These findings that PC has a distinct regenerative property in modulating the function of crucial signaling kinases at the post-translational modification level led us to hypothesize that PC may mimic and advocate the effect of EGF, probably by activating EGFR or cross-talking with this signaling pathway. Therefore, the current study aimed to investigate the combinatory effect of PC and EGF on enhancing human keratinocyte proliferation and survival, and to define the underlying possible mechanism of action. Our report encourages the possible use of PC as an alternative agent or in combination with EGF for accelerating skin wound healing.

## 2. Results

### 2.1. Pinocembrin (PC) Enhances the Effect of Epidermal Growth Factor (EGF) on Stimulating Human Keratinocyte Viability/Proliferation

**Figure 1:**
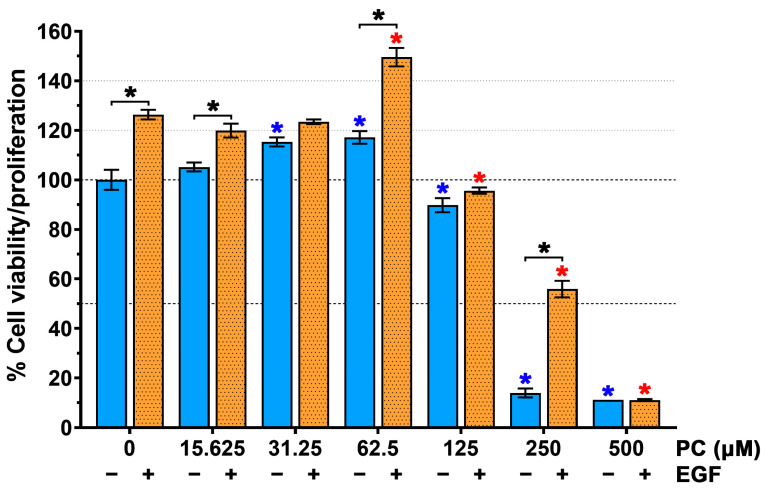
Effects of pinocembrin (PC) on keratinocyte viability determined by MTT assay. Percent viability of cell treated with PC alone at varied concentrations (0–500 µM) or in combination with EGF (1 ng/mL) for 48 h. Values represent mean ± SD of three independent experiments. * *p* < 0.05 indicates significance (blue asterisk (compared to the untreated cells) and red asterisk (compared to EGF-treated cells).

To examine whether PC has potential to enhance the effect of EGF on keratinocyte cell growth and survival, we first performed cell viability assay by MTT. As shown in Figure 1, we found that PC at 31.23 and 62.5 µM increased cell viability of HaCaT cells to 115.31 ± 1.80 and 117.15 ± 2.55%, respectively. Stimulating cells with EGF alone resulted in an increase in cell viability to approximately 126.35 ± 1.97%. Interestingly, co-treatment of cells with EGF and PC at 62.5 µM created a significantly enhanced HaCaT cell viability of 149.6 ± 3.72%. Moreover, as shown in Table 1, the synergy quotient (SQ) calculation of cell viability indicated that a synergistic effect was found when EGF was applied to the treatment of PC at 62.5 μM where the SQ value was 1.14. These results reveal the synergistic effects of combination treatments of EGF with PC at this concentration.

### 2.2. Combinatorial Treatment of PC and EGF Enhances the Healing Rate of Human Keratinocyte Monolayer Wound

**Figure 2:**
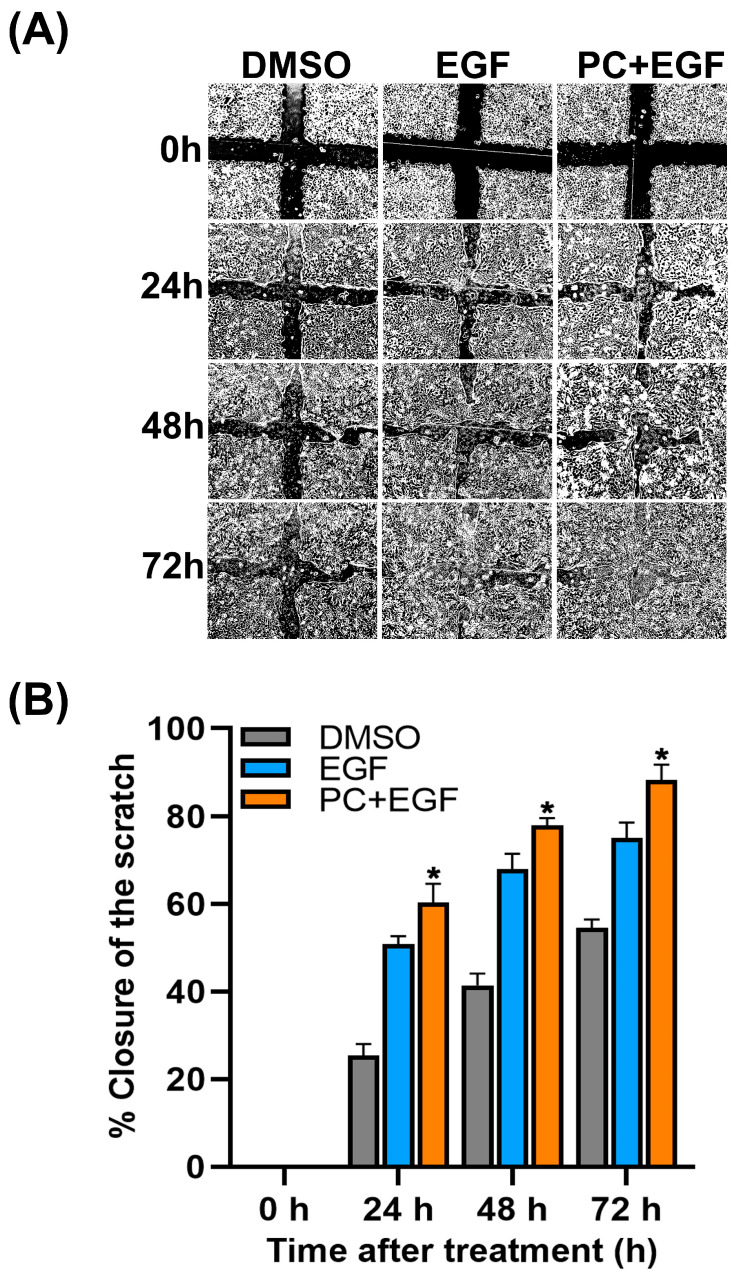
Effects of PC–EGF co-treatment on human keratinocyte monolayer wound-healing rate. (**A**) Scratch monolayer wound-healing assay of human keratinocytes treated with DMSO, EGF, or the combination of PC and EGF at 0, 24, 48, and 72 h. (**B**) Quantitative analysis of the percentage human keratinocytes monolayer wound closure after treatment with different conditions over 72 h. * *p* < 0.05 in comparison to the DMSO (vehicle) control. Data are representatives of three individual replicates.

To elucidate whether PC–EGF co-treatment can enhance the healing rate of epithelial keratinocyte, we performed scratch wound-healing assay. Results obtained from observing under a phase-contrast microscope demonstrated that cells treated with DMSO (vehicle control) showed a base line of monolayer wound closure over the course of 72 h where the scratch wound was still seen (Figure 2A). The closure rate of the scratch wound was quantified to be approximately 20, 40, and 50% at 24, 48, and 72 h, respectively (Figure 2B). In contrast, cells treated with EGF alone increased the wound closure rate to about 50, 60, and 70% at 24, 48, and 72 h, respectively (Figure 2A,B). Notably, treatment of cells with PC alone over the course of 72 h exhibited wound closure-stimulating effects similar to those of EGF-treated cells (data not shown). As we anticipated, cells treated with a combination of PC and EGF significantly increased the rate of wound closure to approximately 60, 80, and 90% at 24, 48, and 72 h, respectively (Figure 2A,B). In addition, we observed that the scratch wound was barely seen at 72 h after PC–EGF co-treatment (Figure 2A). These results indicate that co-treatment of cells with PC plus EGF is more effective than PC or EGF treatment alone in accelerating the closure of the scratch wound. 

### 2.3. Combinatorial Treatment of PC and EGF Enhances the Expansion of Human Keratinocyte Colony by Stimulating An Increase in Cell Number

**Figure 3:**
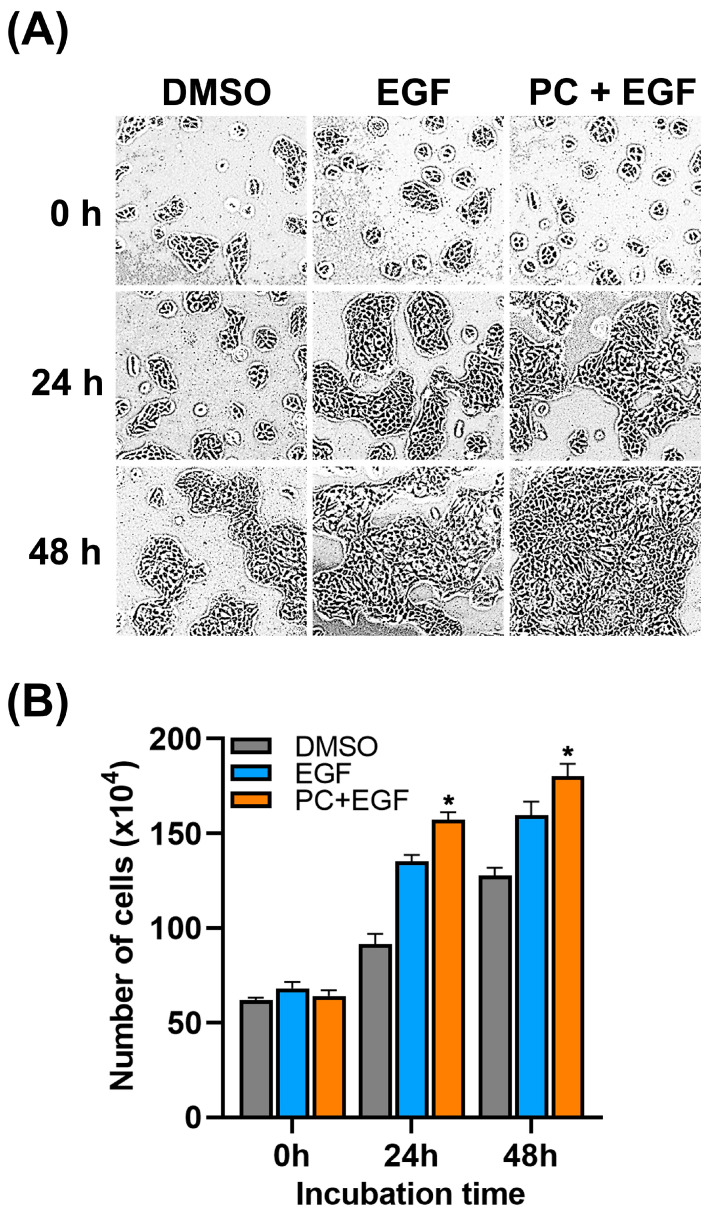
Effects of PC–EGF co-treatment on human keratinocyte proliferation. (**A**) Expansion of keratinocyte colony after treatment with DMSO, EGF, or the combination of PC and EGF over 48 h observed by a phase-contrast microscope (10× magnification). (**B**) Number of cells obtained from direct cell counting at 0, 24, and 48 h post-treatment. Data from three experiments were analyzed and are presented as mean ± SD. * *p* < 0.05 in comparison to the DMSO-treated group.

Based on the results for scratch wound healing shown above, we further examined the stimulating effect of EGF and PC+EGF on the growth of the keratinocyte colony. We observed the expansion of the colony over time after treatment by a phase-contrast microscope along with the total number of cells assayed by direct cell counting at each time point. We found that the colony of the seeded cells at 0 h of treatment was small and distributed evenly (Figure 3A), and the number of cells treated with DMSO, EGF, and PC+EGF was approximately equal (60 × 10^4^ cells) (Figure 3B). The colony of DMSO-treated cells was slightly increased after 24 and 48 h, where the cell number was about 80 × 10^4^ cells and 125 × 10^4^ cells, respectively (Figure 3A,B). The colonies of cells treated with EGF alone at 24 and 48 h post-treatment were obviously increased (Figure 3A), along with an increase in cell number (125 × 10^4^ cells counted at 24 h, and 150 × 10^4^ cells counted at 48 h) (Figure 3B). In addition, we observed that treating cells with PC alone created similar results of colony expansion and cell number to those of EGF-treated cells (data not shown). Interestingly, cells treated with PC+EGF substantially stimulated colony expansion over the course of 48 h (Figure 3A). Consistently, the co-treatment induced a significant increase in cell number after 24 h (150 × 10^4^ cells) and 48 h (175 × 10^4^ cells) (Figure 3B). 

**Figure 4:**
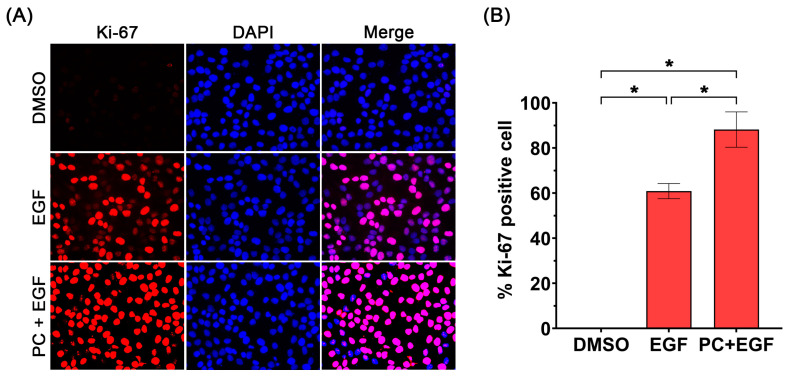
(**A**) Effects of PC–EGF co-treatment on expression of Ki-67 protein (red) in human keratinocytes assayed by immunofluorescence study. The nuclei of the cells (blue) were stained with DAPI and captured at 63× magnification. (**B**) Quantification of the number of Ki-67 positive cells. Data from three experiments were analyzed and are presented as mean ± SD. * *p* < 0.05.

To verify the stimulating effect of PC–EGF combinatorial treatment on cell proliferation, we further detected the proliferative marker, Ki-67, by immunofluorescence study. Results revealed that exposing cells to DMSO in serum-free media did not upregulate the expression of Ki-67 in the nucleus of keratinocytes since individual cells showed a weak basal level of the protein (Figure 4A). In contrast, EGF treatment considerably increased the nuclear expression of Ki-67 (Figure 4A), with the average of Ki-67-positive cells being approximately 60% (Figure 4B). Like EGF treatment, PC alone increased the number of Ki-67-positive cells to around 60% (data not shown). Co-treatment of cells with PC and EGF resulted in a significant increase in the number of keratinocytes with strong Ki-67 expression (almost 90%) (Figure 4A,B).

### 2.4. Combinatorial Treatment of PC and EGF Enhances the Activation of ERK1/2 and Akt 

**Figure 5:**
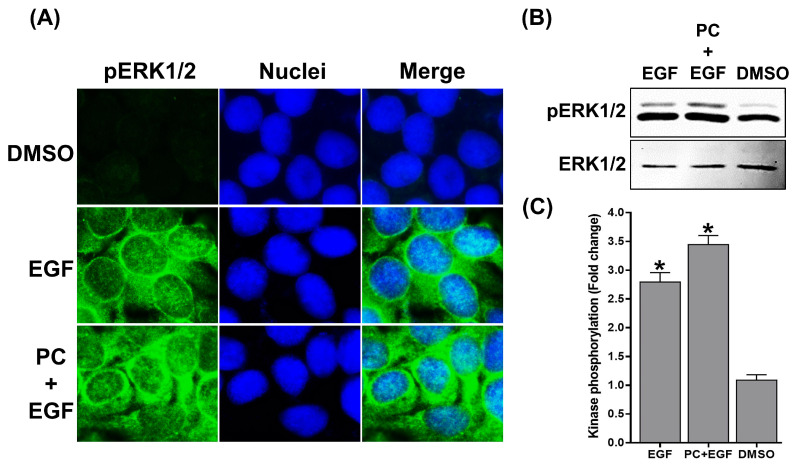
(**A**) Immunofluorescence study detecting the phosphorylation of ERK1/2 upon different treatments (DMSO, EGF, or the combination of PC and EGF). Cells were stained with DAPI for nuclear visualization (blue). Micrographs were photographed by a fluorescent microscope (100× magnification). (**B**) Western blot analysis detecting the phosphorylation of ERK1/2 in the cell lysates upon different treatments (DMSO, EGF, or the combination of PC and EGF). (**C**) Quantification of immunoreactive bands of phosphorylated ERK1/2. Data from three experiments were analyzed and presented as mean ± SD. * *p* < 0.05 compared to DMSO-treated cells.

We further attempted to elucidate whether PC and EGF exert a similar mechanism of action in stimulating growth and survival of keratinocytes. To prove this, we focused on the activation status of major signal transduction pathways responsible for cell growth and survival. We found that, in comparison to DMSO-treated cells, treatment with EGF alone visibly increased the level of pERK1/2 in individual cells (Figure 5A) while PC–EGF co-treatment drastically increased the intracellular signal of the kinase (Figure 5A). Data from Western blot analysis verified that the immunoreactive band intensity of pERK1/2 in EGF-treated cells was significantly increased to approximately 2.5-fold compared to that of DMSO-treated cells (Figure 5B,C), and a similar level of increased pERK1/2 was seen in cells treated with PC alone (data not shown). As predicted, cells exposed to a combination of PC and EGF showed significantly enhanced phosphorylation of ERK1/2 to approximately 3.5-fold in comparison to that of DMSO-treated cells (Figure 5B,C).

**Figure 6:**
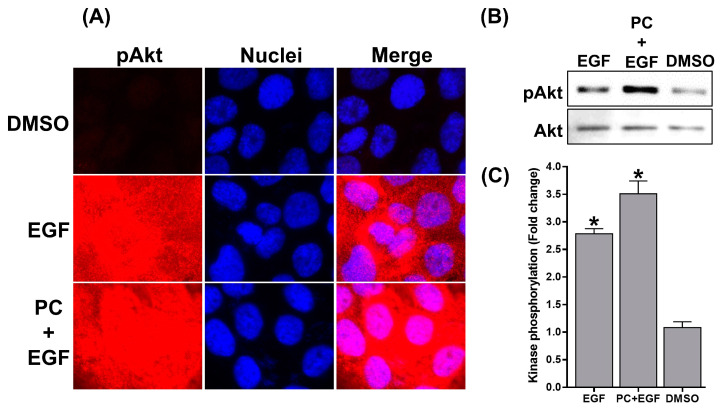
(**A**) Immunofluorescence study detecting the phosphorylation of Akt upon different treatments (DMSO, EGF, or the combination of PC and EGF). Cells were stained with DAPI for nuclear visualization (blue). Micrographs were photographed by a fluorescent microscope (100× magnification). (**B**) Western blot analysis detecting the phosphorylation of Akt in the cell lysates upon different treatments (DMSO, EGF, or the combination of PC and EGF). (**C**) Quantification of immunoreactive bands of phosphorylated Akt. Data from three experiments were analyzed and are presented as mean ± SD. * *p* < 0.05 compared to DMSO-treated cells.

In line with the results from pERK1/2 detection, a survival marker, Akt, was clearly detected in EGF-treated cells (Figure 6A). The level of EGF-induced pAkt was quantified to be around 2.8-fold (Figure 6B,C), which was like that of PC-treated cells (data not shown). Consistent with the observation of pERK1/2, simultaneous exposure of cells with PC and EGF induced pAkt more strongly than did EGF alone (Figure 6A,B), where pAkt was increased to around 3.5-fold (Figure 6C). 

### 2.5. PC Does Not Transactivate Epidermal Growth Factor Receptor (EGFR)

**Figure 7:**
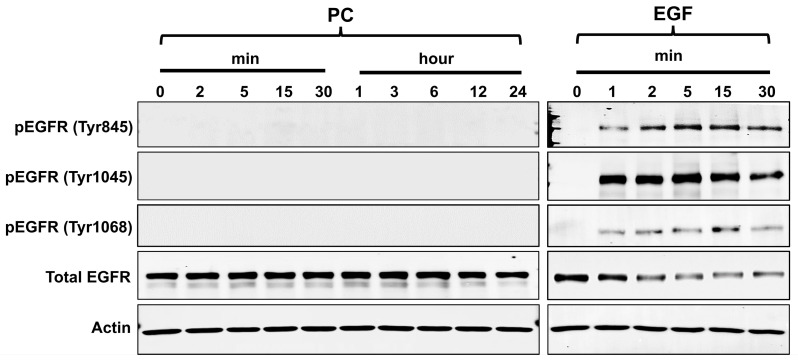
Western blot analysis detecting the phosphorylation of epidermal growth factor receptor (EGFR) at tyrosine (Tyr) residue 845, 1045, and 1068 and the total form of EGFR in the cell lysates upon different treatments (DMSO, PC, EGF, or the combination of PC and EGF) at various time points. β-actin was used as a loading control.

Since we observed that PC–EGF co-treatment enhanced cell proliferation and survival through activating ERK1/2 and Akt, it is possible that PC may be a small molecule that mimics the activity of EGF, which acts on its receptor. To address this hypothesis, we determined the activation of the epidermal growth factor receptor (EGFR) upon EGF or PC stimulation at various time points. Undoubtedly, stimulation of cells with EGF rapidly activated strong phosphorylation of EGFR on tyrosine (Tyr) 845, 1045, and 1068 over the course of 30 min (Figure 7). Consistent with a significant increase in EGFR phosphorylation, the total form of EGF receptor was gradually reduced over time, indicating that the activated receptor is being degraded (Figure 7), which is a general negative feedback regulation in response to the activation of EGFR. In contrast, cells exposed to PC over 30 min did not stimulate phosphorylation of EGFR at any tyrosine residue, and total EGFR was not degraded (Figure 7). Similarly, treatment of cells with PC for extended periods of time (1, 3, 6, 12, and 24 h) demonstrated that PC did not stimulate EGFR phosphorylation (Figure 7).

**Figure 8:**
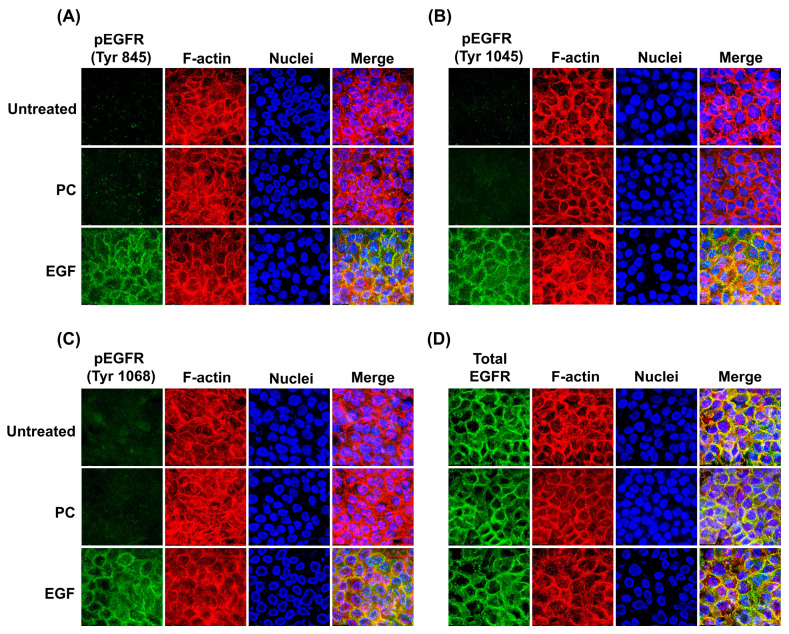
Immunofluorescence study detecting the phosphorylation of EGFR at Tyr845 (**A**), Tyr1045 (**B**), and Tyr1068 (**C**), and total EGFR (**D**), upon different treatments (untreated (UT), PC, or EGF). Cells were counterstained with DAPI for nuclear visualization (blue) and Phalloidin for filamentous actin (F-actin) (red). Micrographs were photographed by a fluorescent microscope (100× magnification).

To verify the results from Western blot analysis, we visualized the activation of EGFR in individual cells by immunofluorescence study. Results confirmed that EGF-treated cells exhibited strong phosphorylation of EGFR at tyrosine 845 (Figure 8A), 1045 (Figure 8B), and 1068 (Figure 8C), where the signal was mainly visualized at areas of the cell-to-cell contact. However, cells exposed to PC showed a negative signal of pEGFR, while the total EGFR was detected to be significant, with the staining pattern indistinguishable from that of the untreated or EGF-treated cells (Figure 8D).

## 3. Discussion

During the natural wound-healing process, many growth factors, including epidermal growth factor (EGF), are secreted at the wound site to stimulate cell proliferation, differentiation, and survival [39,40,41]. Nevertheless, this response may be delayed in the elderly, especially those who have chronic wounds where the wound may fail to achieve complete re-epithelialization in the appropriate timeframe due to a decline in endogenous growth factor levels or activity at the wound site [41,42]. Therefore, there have been many attempts to utilize the biological benefits of human recombinant EGF in medical practice for improving wound healing. However, EGF is a large molecule with a short half-life of action [43,44,45] and is degraded by biofilm proteases [18]. Moreover, the cost of producing pure human recombinant EGF is still expensive. Hence, discovering a stable molecule that mimics the action of EGF or cross-talks with the epidermal growth factor receptor (EGFR) signaling pathway may help enhance the healing effect of EGF. Since our previous study reported that pinocembrin (PC) stimulated the activation of ERK1/2 and PI3K/Akt and promoted human keratinocyte proliferation and survival [38], the current study attempted to investigate whether this molecule enhances the effects of EGF on accelerating the growth and survival of human keratinocytes.

We initially examined the effect of PC–EGF co-treatment on cell viability of human keratinocytes. Our results demonstrated that treatment of cells with PC alone increased keratinocyte cell viability in a concentration-dependent manner, and we observed that the synergy quotient (SQ) of cell viability was found when we combined EGF with PC at 62.5 μM. This result indicates that PC at this specific concentration can synergize with EGF to enhance keratinocyte cell viability, which means that co-treatment of PC–EGF stimulates human keratinocyte viability/proliferation more effectively than does PC or EGF treatment alone. This finding led us to hypothesize that combinatorial treatment of PC and EGF may accelerate the healing rate of the keratinocyte monolayer. Data from scratch wound-healing assay revealed the substantial effect of PC–EGF co-treatment on enhancing the keratinocyte monolayer over time in comparison to that of DMSO, PC, or EGF treatment. These results shed light on the advantage of combining PC with EGF for enhancing the rate of scratch wound closure. One possible explanation of this phenomenon was proved to be caused by a drastic increase in keratinocyte proliferation, since PC–EGF co-treatment significantly activated an increase in number of cells over time. In accordance with this finding, there was a remarkable increase in the number of cells expressing Ki-67 protein in the nucleus compared to DMSO-treated, PC-treated, or EGF-treated cells. These data verify that PC–EGF co-treatment enhances keratinocyte monolayer healing, in part through stimulating cell proliferation, since Ki-67 has been reported to be one of important cellular markers for proliferation [46]. To further elucidate the possible mechanism of action underlying the wound-healing-promoting effects of PC–EGF co-treatment on keratinocyte, we directed our focus on the epidermal growth factor receptor (EGFR) signaling pathway, which is an important cascade that stimulates epidermal cell growth, proliferation, and survival [16]. We first detected the activation status of MAPK/ERK and PI3K/Akt since the activation of these molecules ultimately turns on certain gene expression involved in cell growth and survival [12,47]. As we anticipated, treatment with EGF alone activated its downstream ERK1/2 and Akt. Interestingly, our additional results revealed that PC treatment alone also activated these two kinases with an activation pattern similar to that of EGF treatment. However, combination of EGF and PC significantly enhanced the activation of ERK1/2 and Akt. These results strongly suggest that PC can stimulate keratinocyte proliferation and migration, as well as enhance the effect of EGF in part through activating ERK1/2 and Akt phosphorylation. In line with the previous studies, many substances have been reported to promote proliferation and migration of human keratinocytes via inducing the MAPK and PI3K/Akt signaling pathways. For instance, a short peptide AES16-2M could accelerate animal skin wound healing via increasing keratinocyte and fibroblast migration [48]. Another human peptide, cathelicidin LL-37, was reported to possess wound-healing-promoting activities through activating many signaling pathways, including MAPK and PI3K/Akt [49]. The activation of MAPK and PI3K/Akt is known as a molecular hallmark of the EGFR signaling pathway [12,47] which is vital in promoting the growth, proliferation, and survival of keratinocytes [14,15,16]. Certain natural compounds, including piperonylic acid isolated from black (*Piper nigrum* L.) and long (*Piper longum* L.) peppers [22] and isoprocurcumenol found in *Curcuma longa* L. [23], have been reported to support or stimulate human keratinocyte growth and survival through activating EGFR and its downstream ERK1/2 and Akt kinases. Our previous observation that PC can activate MAPK and PI3K/Akt [38] led us to think about the possibility that PC may be another natural compound that can activate EGFR. We thus further explored this hypothesis. Surprisingly, data from Western blot analysis and immunofluorescence study clearly showed that while EGF rapidly and robustly stimulated EGFR phosphorylation at tyrosine 845, 1045, and 1068, PC did not activate phosphorylation of EGFR at any tyrosine residue. These results indicate that PC-induced phosphorylation of ERK1/2 and Akt is not mainly mediated through the transactivation of EGFR. PC may exert a different mechanism to activate ERK1/2 and Akt phosphorylation. It has been reported that peptide LL-37 could mediate the activation of the MAPK and PI3K/Akt signaling events through the induction of G-protein-coupled receptor, in addition to its ability to transactivate EGFR. Therefore, one possible mechanism for ERK1/2 and Akt activation by PC that enhanced the effects of EGF may be mediated through the stimulation of G-protein-coupled receptor. Our idea is clearly supported by a recent study that discovered that PC is a GPR120 ligand, which shares the same pocket occupied by the known GPR120 agonist (TUG-891), and that PC binding to GPR120 led to a rapid stimulation of key signaling events including ERK1/2 and Akt [50]. To clearly understand the accurate signal transmission induced by PC, study at the structural level including the redesign of a functional kinase–substrate interaction [51] may be required. This type of experimental model may provide beneficial information explaining whether PC specifically interacts with and activates certain intracellular kinases responsible for enhancing the growth and survival of human keratinocyte.

Taken together, we discovered that co-treatment of PC and EGF enhances human keratinocyte growth and survival more effectively than does single treatment of PC or EGF. The possible reason underlying the synergistic effects of PC–EGF combinatorial treatment may be explained by the fact that the activation of a proliferative marker, ERK1/2, and a survival marker, Akt, is actively augmented via two unique signaling cascades. One is the EGFR signaling induced by EGF, the other is GPR120 induced by PC. Our data may offer an additional approach to the management of skin wounds, especially in individuals who exhibit low sensitivity to single-agent treatment.

## 4. Conclusions

**Figure 9:**
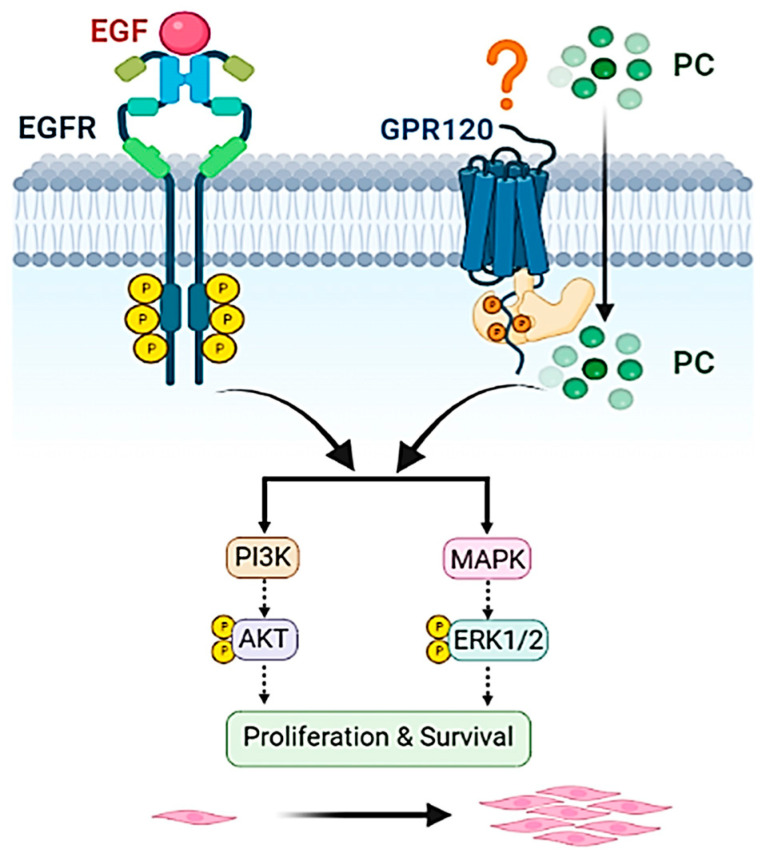
A proposed model explaining the possible effects of PC–EGF co-treatment on the growth and survival of human keratinocytes. The schematic picture was designed using BioRender.com (accessed on 16 June 2023).

As shown in the schematic in Figure 9, we demonstrated that PC actively enhanced the effects of EGF on stimulating human keratinocyte proliferation and survival. The responsible molecular mechanism of action of PC was proven to be independent of the activation of EGFR and supported by another group, possibly through the activation of G-protein-coupled receptor, GPR120. Discovering that PC has a unique activation pattern, yet maintains the ability to cross-talk with the downstream molecular players of the EGFR, provides the possibility to develop and clinically apply PC as a monotherapy or in combination with EGF for effectively enhancing wound healing, especially in cases in which the wound is less responsive to currently available standard treatments.

## 5. Materials and Methods

### 5.1. Cell Line and Cell Culture

Human keratinocyte cell line (HaCaT) was commercially obtained (CLS Cell Lines Service, Eppelheim, Baden-Wurttemberg, Germany). This cell line was originally derived and isolated from a 62-year-old male patient [52]. HaCaT cells were maintained using complete Dulbecco’s modified eagle medium (DMEM) which contained 10% fetal bovine serum (FBS) consisting of sufficient nutrients to induce cell proliferation and differentiation [53]. Antibiotics (100 U/mL penicillin and 100 g/mL streptomycin) were added to the media to prevent contamination. Subculture of the cells was performed and complete DMEM was replaced every 3–4 days.

### 5.2. Epidermal Growth Factor (EGF) and Pinocembrin (PC)

Recombinant human EGF was purchased from ImmunoTools (Friesoythe, Niedersachsen, Germany). The 500 µg/mL stock of rhEGF was prepared according to the company’s instructions, further diluted to 10 µg/mL, and stored at −20 °C. PC was purchased from MilliporeSigma (Burlington, MA, USA). Pinocembrin (purchased from MilliporeSigma) stock solution was prepared in dimethyl sulfoxide (DMSO) to be 195.12 mM and stored at −20 °C.

### 5.3. Test of Cell Viability

In 96-well plates, HaCaT cells were seeded at 1.0 × 10^4^ cells/well (200 µL) in complete DMEM and incubated for 24 h. Then, cells were treated with PC alone at various concentrations (0–500 µM) or PC in combination with EGF at 1 ng/mL for 48 h. Then, cells were cultured in 200 µL of complete DMEM containing MTT reagent (0.4 mg/mL) for 1 h. After discarding the culture supernatant, the intracellular MTT crystal was solubilized by adding 100 µL of 100% DMSO. The colorimetric signal was measured at 570 nm by a microplate reader (BioTek Instruments, Winoouski, VT, USA).

### 5.4. Monolayer Wound-Healing Activity Assay

HaCaT cells, at a seeding density of 1 × 10^5^ cells/well (500 μL), were cultured in 24-well plates in complete DMEM and allowed to proliferate until they reached 95–100% confluency. Cells were then serum-starved for 24 h in DMEM without the presence of FBS. A SPLScar™ Scratcher (SPL Life Sciences, Gyeonggi-do, Republic of Korea) was used to scrape the cell monolayer (in a vertical and horizontal scratch lines) to create an in vitro cell monolayer wound model in each well. The cells were washed once with sterile PBS to clear any debris and floating cells, and then the well was refilled with serum-free DMEM or DMEM containing DMSO (vehicle control), PC at 62.5 μM, EGF at 1 ng/mL, or the combination of PC and EGF. HaCaT monolayer wound closure was monitored under a phase-contrast microscope at 0 h, 24 h, 48 h and 72 h. Percent closure of the scratch at each time point for different treatment conditions was then quantified. Each treatment was performed in triplicate, and each experiment was repeated at least three times.

### 5.5. Colony Expansion and Cell Counting Assay

HaCaT cells were seeded in 24-well plates in complete DMEM and cultured overnight. Cells were then serum-starved for 24 h in DMEM without the presence of FBS. Cells were then treated with several different treatment conditions (DMSO, PC alone, EGF alone, or a combination of PC and EGF in serum-free DMEM). The size of the HaCaT colony was captured at 0 h, 24 h, and 48 h at 10× magnification using an Axio Vert.A1 microscope (Carl Zeiss Suzhou Co., Ltd., Suzhou, China). Moreover, after the image of the colony at each time point was recorded, the total number of cells was counted using a CellDrop™ Automated Cell Counter (DeNovix Inc., Wilmington, DE, USA). 

### 5.6. Immunofluorescence Staining

To localize and investigate molecular signaling of cell proliferation and cell survival, immunofluorescence staining was performed. Cover slips were cleaned using 95% ethanol and flame. Then, 500 μL of DMEM-containing cells were added to each well of 24-well plates containing a sterile glass cover slip, and cells were allowed to reach confluence. For Ki-67 detection, cells were treated with several different conditions as explained above for 24 h. Next, fixation was performed by adding 4% paraformaldehyde/PBS solution to each well and incubating for 15 min at room temperature (RT). After washing with PBS three times, 0.3% TritonX-100 was added for cell permeabilization for 5 min at RT. A blocking step was performed by adding 1% BSA/PBS to each well for 1 h at RT. The cover slips were then incubated with rabbit anti-human Ki-67 antibody for 24 h at 4 °C in a moist chamber. The next day, the coverslips were washed three times and incubated with goat anti-rabbit IgG (Alexa594-conjugated) (Thermo Fisher Scientific, Waltham, MA, USA) for 2 h at RT in a moist chamber, in the dark. The nucleus of cells was counterstained with DAPI at 1 µg/mL (MilliporeSigma). Then, coverslips were washed three times with PBS, one more time with sterile deionized water, and mounted with Fluoromount-G (Southern Biotech, Bermingham, AL, USA). The fluorescent signal of a nuclear Ki-67 protein and DAPI were visualized at 100× magnification with a fluorescence microscope Axio Vert.A1 (Carl Zeiss Suzhou Co., Ltd., Suzhou, China) with the Zen version 2.6 (blue edition) software for the Zeiss Axiocam 506 color microscope camera being used to capture and analyze the images. For detecting the early signaling pathways, cells were seeded on the coverslip as explained and cultured in complete DMEM for 24 h. The old media were replaced with serum-free media, and cells were incubated for 24 h before treatment. Cells were treated with DMSO, PC, EGF, or a combination of PC and EGF for 15 min (for detecting the phosphorylation of ERK1/2 and Akt) and 1 min (for detecting the phosphorylation of epidermal growth factor receptor (EGF)) before being subjected to fixation, permeabilization, and blocking as explained above. The coverslips were then incubated for 24 h at 4 °C with specific primary antibodies, which included rabbit anti-pERK1/2 antibody, rabbit anti-pAkt antibody, rabbit anti-pEGFR (tyrosine (Tyr) 845) antibody, rabbit anti-pEGFR (tyrosine (Tyr) 1045) antibody, rabbit anti-pEGFR (tyrosine (Tyr) 1068) antibody, and rabbit anti-total EGFR antibody. Cells were then incubated with a secondary antibody conjugated with Alexa488 or Alexa594 mixed with 1 μg/mL of DAPI for 2 h. In some experiments, filamentous actin (F-actin) was detected with DyLight-594-Phalloidin. Primary antibodies, DAPI, and DyLight-594-Phalloidin were purchased from Cell Signaling Technology (Boston, MA, USA). Visualization and image processing were performed as explained above.

### 5.7. Immunoblotting Assay

To investigate proliferation and survival signaling in human keratinocytes after treatment with different treating conditions, Western blotting was performed. Specifically, HaCaT cells at a density of 1.5 × 10^5^ cells/well were seeded in 24-well plates and then serum-starved overnight. Keratinocytes were treated with various conditions for different time points. After that, 1× reducing Laemmli buffer (containing sodium dodecyl sulfate (SDS) and 2-mercaptoethanol (2-ME)) was added to each well to make cell lysates. A dry bath incubator (heating block) at 95 °C was used to heat the lysates for 5 min, and the sample lysates were subjected to sodium dodecyl sulfate polyacrylamide gel electrophoresis (SDS-PAGE) and electroblotting onto the polyvinylidene fluoride (PVDF) membrane. For blocking nonspecific binding, the membrane was incubated with 5% bovine serum albumin (BSA) for 1 h; then, the membrane was incubated overnight with specific primary antibodies (Cell Signaling Technology). Those antibodies were a rabbit anti-pERK1/2 antibody, a rabbit anti-ERK1/2 antibody, a rabbit anti-pAkt antibody, a rabbit anti-Akt antibody, a rabbit anti-pEGFR (Tyr845) antibody, a rabbit anti-pEGFR (Tyr1045) antibody, a rabbit anti-pEGFR (Tyr1068) antibody, a rabbit anti-total EGFR antibody, and a mouse anti-β-actin antibody. Then, membranes were washed with TBS-Tween and incubated with appropriate secondary antibodies (LI−COR Biosciences, Lincoln, NE, USA) for 2 h. The positive bands were visualized using an Odyssey^®^ CLx Imaging System (LI−COR Biosciences, Lincoln, NE, USA).

### 5.8. Statistical Analysis

All experimental data were analyzed by one-way ANOVA followed by the Tukey-Kramer multiple comparisons test on RAW data reads using GraphPad Prism version 9.0.0 (121) (GraphPad Software Inc., San Diego, CA, USA) and represented as the mean ± standard deviation (SD). * *p* values less than 0.05 were considered statistically significant. Each experiment was performed at least three times.

### 5.9. Synergy Quotient Calculation for Synergism

The synergism quotient (SQ) was determined by deducting the baseline values from all treatments and then dividing the net effect of the combination [X + Y] by the sum of individual effects [X] + [Y]. SQ greater than 1.0 indicates a synergistic effect.

## Figures and Tables

**Table 1:** Synergism quotient of EGF on the PC-induced increase in cell viability/proliferation of human keratinocyte.

Treatment	Cell Viability/Proliferation (%)			Growth Rate	SQ Value
EGF	126.35	±	1.97	26.35	
15.625 µM PC	105.20	±	1.81	5.20	
EGF + 15.625 µM PC	119.90	±	2.82	19.90	0.63
31.25 µM PC	115.31	±	1.80	15.31	
EGF + 31.25 µM PC	123.44	±	0.97	23.44	0.56
62.50 µM PC	117.15	±	2.55	17.15	
EGF + 62.50 µM PC	149.60	±	3.72	49.60	1.14

## Data Availability

All data, tables and figures are original and are available in this article.
